# Extinction and dawn of the modern world in the Carnian (Late Triassic)

**DOI:** 10.1126/sciadv.aba0099

**Published:** 2020-09-16

**Authors:** Jacopo Dal Corso, Massimo Bernardi, Yadong Sun, Haijun Song, Leyla J. Seyfullah, Nereo Preto, Piero Gianolla, Alastair Ruffell, Evelyn Kustatscher, Guido Roghi, Agostino Merico, Sönke Hohn, Alexander R. Schmidt, Andrea Marzoli, Robert J. Newton, Paul B. Wignall, Michael J. Benton

**Affiliations:** 1 School of Earth and Environments, University of Leeds, Leeds, LS2 9JT, UK.; 2 State Key Laboratory of Biogeology and Environmental Geology, School of Earth Sciences, China University of Geosciences Wuhan, Wuhan, China.; 3 MUSE–Science Museum, 38122 Trento, Italy.; 4 School of Earth Sciences, University of Bristol, Bristol BS8 1RJ, UK.; 5 GeoZentrum Nordbayern, Universität Erlangen-Nürnberg, 91054 Erlangen, Germany.; 6 Department of Palaeontology, University of Vienna, 1090 Wien, Austria.; 7 Department of Geosciences, University of Padova, 35131 Padova, Italy.; 8 Department of Physics and Earth Sciences, University of Ferrara, 44100 Ferrara, Italy.; 9 School of Natural and Built Environment, Queen’s University Belfast, Belfast, BT7 1NN, Northern Ireland, UK.; 10 Museum of Nature South Tyrol, 39100 Bozen/Bolzano, Italy.; 11 Department of Earth and Environmental Sciences, Paleontology & Geobiology, Ludwig-Maximilians-Universität München, 80333 München, Germany.; 12 SNSB-Bayerische Staatssammlung für Paläontologie und Geologie, 80333 München, Germany.; 13 Institute of Geosciences and Earth Resources (IGG-CNR), 35131 Padova, Italy.; 14 Leibniz Centre for Tropical Marine Research (ZMT), 28359 Bremen, Germany.; 15 Department of Physics and Earth Sciences, Jacobs University Bremen, 28759 Bremen, Germany.; 16 Department of Geobiology, University of Göttingen, 37077 Göttingen, Germany.

## Abstract

The Carnian Pluvial Episode (Late Triassic) was a time of global environmental changes and possibly substantial coeval volcanism. The extent of the biological turnover in marine and terrestrial ecosystems is not well understood. Here, we present a meta-analysis of fossil data that suggests a substantial reduction in generic and species richness and the disappearance of 33% of marine genera. This crisis triggered major radiations. In the sea, the rise of the first scleractinian reefs and rock-forming calcareous nannofossils points to substantial changes in ocean chemistry. On land, there were major diversifications and originations of conifers, insects, dinosaurs, crocodiles, lizards, turtles, and mammals. Although there is uncertainty on the precise age of some of the recorded biological changes, these observations indicate that the Carnian Pluvial Episode was linked to a major extinction event and might have been the trigger of the spectacular radiation of many key groups that dominate modern ecosystems.

## Teaser

The roots of modern-style ecosystems go back to the Carnian (ca. 233 Ma), a time of global climate change and extinction.

## INTRODUCTION

**Fig. 1:**
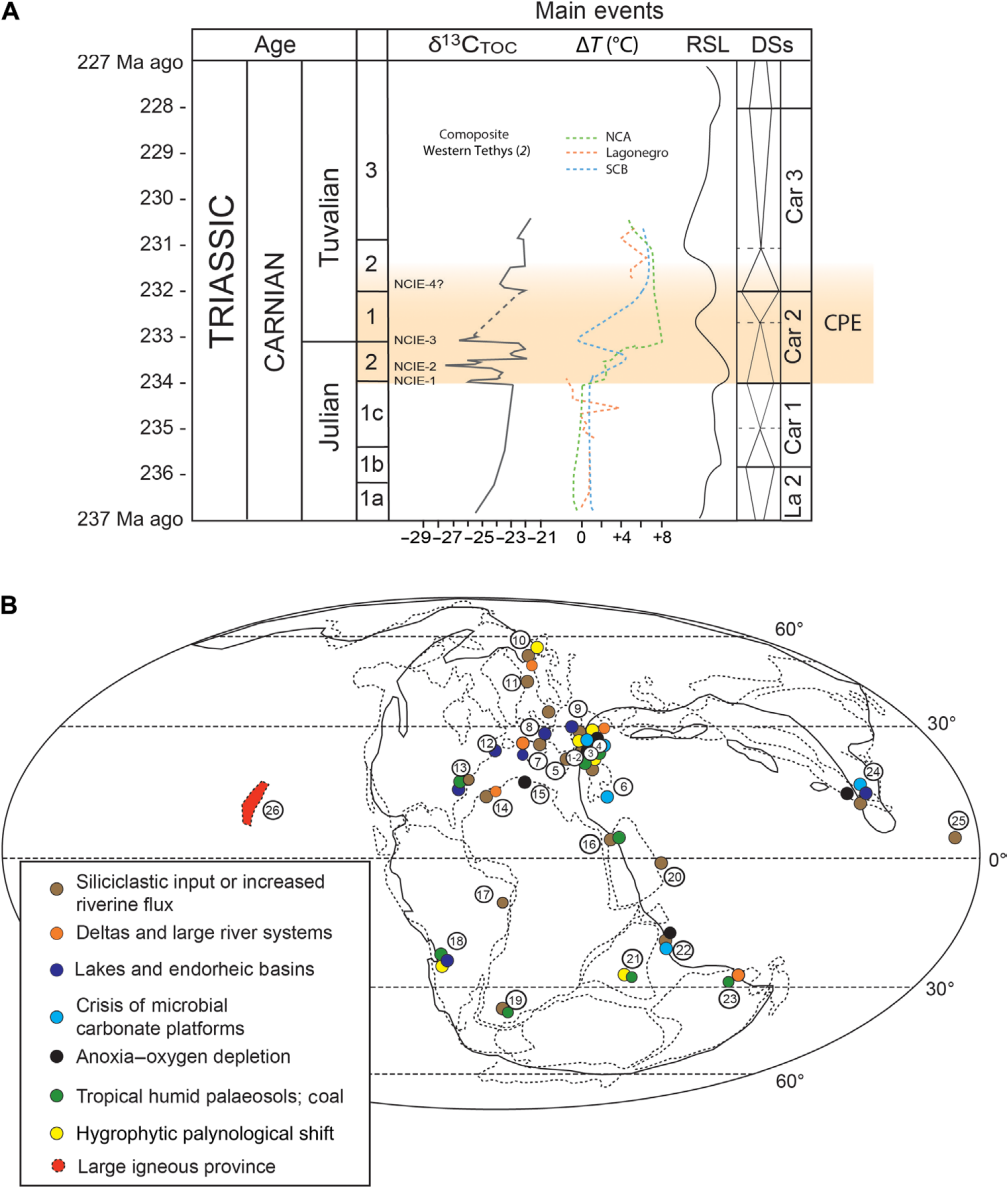
Environmental and geochemical changes of the CPE. (**A**) Age of the CPE, composite organic carbon-isotope curve through the Carnian (*2*), temperature changes reconstructed from oxygen-isotope data from conodont apatite (*7*), relative sea level changes (RSL), and depositional sequences (DSs). The gap in data in the lower Tuvalian is centered on a global sea level low, implying that most shallow water successions have a hiatus there. The reader should refer to (*2*) and (*7*) for a detailed discussion of the δ^13^C and temperature composite schematic records. NCA, Northern Calcareous Alps; SCB, South China Block. (**B**) Palaeogeography during the Carnian and location of the data indicating environmental changes during the CPE [modified after (*2*)]. Localities: (1) Dolomites (Italy), (2) Julian Alps (Italy), (3) Transdanubian Range (Hungary), (4) Northern Calcareous Alps (Austria), (5) Lagonegro Basin (Italy), (6) Antalya (Turkey), (7) Iberia (Spain), (8) United Kingdom, (9) Central European Basin, (10) Barents sea, (11) Jameson Land, (12) Fundy Basin (Canada), (13) Richmond and Taylorsville basins (United States), (14) Essaouira Basin, (15) Jeffara Basin, (16) Levant Basin (Jordan, Israel), (17) Brasil, (18) Ishigualasto and Cujo basins (Argentina), (19) Karoo Basin (South Africa), (20) Oman, (21) Rewa Basin (India), (22) Spiti (India), (23) Carnarvon Basin (Australia), (24) Nanpanjiang Basin (China), (25) Panthalassa (Japan), and (26) Wrangellia and Panthalassa (Canada and United States).

The Carnian, Late Triassic (Fig. 1), marks a time of profound changes to life, in the ocean and on land. During this stage, a major climate change occurred, namely, the Carnian Pluvial Episode (CPE; Fig. 1). The CPE occurred around 234 to 232 million years (Ma) ago. Its most marked characteristic was a remarkable enhancement of hydrological cycling marked by four episodes of increased rainfall, as indicated by diverse sedimentary and paleontological data (*1*–*3*). Repeated C cycle perturbations, evidenced by sharp negative C-isotope excursions, coincided with global environmental changes and climate warming, suggesting a cause-and-effect relationship (*2*, *4*–*7*).

Recent field studies show that the CPE was a global phenomenon (*5*, *8*); geochemical data suggest that global warming triggered environmental and biotic changes and, along with a small number of radioisotopic ages coupled with biostratigraphic correlation, suggest a possible link to the eruption of the Wrangellia Large Igneous Province (LIP) (*2*, *4*–*7*, *9*, *10*). Fossil finds have clarified the timing of extinctions and radiations of many groups during the CPE (*10*–*14*); recent studies (*10*, *14*) show that, while dinosaurs originated in the Early to Middle Triassic, they remained rare and at low diversity and only radiated explosively during the CPE.

Paleobiologists have noted a biological change around the time of the CPE since the 1960s, but it has been difficult to identify this event as a major biological turnover for two reasons: lack of confidence in dating and correlation of marine and terrestrial sediments and the occurrence of the CPE in the middle of a stratigraphic stage, which made it undetectable in most database analyses that used stage time bins with durations of 10 to 11 Ma [e.g. (*15*)]. Nonetheless, two mass extinctions in the Late Triassic, one the long-recognized end-Triassic event and another in the Carnian, were identified over 30 years ago (*16*). The Carnian event stood out in the epoch-level datasets for ammonoids and tetrapods, with an extinction peak in the early Carnian for the former and the late Carnian for the latter.

Here, we review the evidence of a major change in ecological community structure during the Carnian, and in particular, we discuss the temporal links of these biological changes with the CPE and the role of major volcanic eruptions and associated climate change as the most plausible trigger. We summarize the recent geochemical evidence for multiple C cycle perturbations and global warming during the CPE and the associated observed changes in the different depositional environments. The marine and terrestrial fossil records are reviewed in light of recent dating and stratigraphic correlations. In addition, we have reviewed published paleontological databases of marine generic occurrences at the substage level and, when possible, modified the age of the entries to biozone level to better assess the scale of the Carnian crisis. We show that existing data indicate that the CPE was a major extinction event that was followed by an explosive diversification of important organisms in the sea and on land that now play a key role in modern ecosystems.

## AGE CONSTRAINTS

The Carnian is the basal stage of the Late Triassic. Its lower boundary is dated at ca. 237 Ma ago, based on U-Pb radiometric dating of single-crystal zircons from a tuff layer within a section having strong biostratigraphic constraints (*17*, *18*), and its upper boundary at ca. 227 Ma ago, based on magnetostratigraphic correlations between the marine successions of Tethys and the astrochronological time scale of the continental Newark Basin (Fig. 1) (*19*). The stage is subdivided into the Julian (early Carnian) and Tuvalian (late Carnian) substages; the Julian-Tuvalian boundary occurs at ca. 233 Ma ago (*19*).

The onset of the CPE is readily recognizable and synchronous in many geological settings thanks to ammonoid, conodont, and sporomorph biostratigraphic dating. It coincides with the first appearance of the ammonoid genus *Austrotrachyceras* in the Julian (*1*, *4*, *5*, *7*, *20*, *21*). The end of the CPE is less well studied and poorly defined in most locations, but it is usually placed at the base or within the Tuvalian 2 on the basis of sedimentological (e.g., end of terrigenous sediment supply) and chemostratigraphic (last C-isotope excursion) evidence (*2*). Cyclostratigraphy of marine successions of the South China Block and of continental successions of the Wessex Basin (United Kingdom) gives a duration of the CPE of approximately 1.2 Ma (*6*), although integrated stratigraphy (biostratigraphy and magnetostratigraphy) suggests a longer duration of approximately 1.6 to 1.7 Ma (*10*).

## OVERVIEW OF THE ENVIRONMENTAL CHANGES DURING THE CPE

### Sedimentary changes

Major changes in sedimentary records from deep water to terrestrial settings are recorded during the CPE. The changes, although locally variable, present some common characteristics that can be traced extensively (Fig. 1). These can be summarized as (i) profound transformation or interruption of carbonate sedimentation and increase in terrigenous input into marine basins and (ii) shifts in sedimentation indicating a major variation of the hydrological regime in terrestrial depositional settings. In general, the sedimentary record suggests an enhancement of the hydrological cycling during the CPE. In the next paragraphs, we will briefly summarize such changes.

#### Deep water

Records of the CPE in the deep ocean (bathyal zone or deeper) are scarce because most Triassic oceanic crust and sediment has been subducted, and the accreted fragments are often deformed and thermally altered. Nonetheless, a climatic signal can be deduced from the successions in Japan (Fig. 1), where the CPE is recorded as a major change in the composition of wind-blown clay minerals (the arrival of smectite), formed on land following increased global humidity (*22*). Western Tethyan Middle-Late Triassic deep-water successions sit on continental crust and comprise cherty limestone, with one interruption of clay and radiolarite at the CPE in the Lagonegro Basin in Southern Italy (Fig. 1) (*11*). This interruption in deep-water carbonate sedimentation, which occurs in the late Julian and lasts until the Tuvalian 2, is unique in the Late Triassic and suggests a temporary rise of the carbonate compensation depth and/or the shutdown of a carbonate supply from adjacent shallow water platforms at the CPE (*11*), as it is explained in the following paragraph.

#### Shallow water

During the CPE, a major change in shallow carbonate systems saw the shift from microbially dominated carbonate-producing ecosystems (carbonate factories) to less productive metazoan-dominated ecosystems, with the appearance of modern-style scleractinian coral reefs (see also the discussion below on the CPE marine biological changes). This coincided with the first C-isotope excursion at the onset of the CPE (*5*, *23*). The crisis of the Middle Triassic–early Carnian carbonate factories during the CPE was at least Tethys wide (*2*, *7*, *20*, *24*). The other major change in the Carnian was a widespread increase of terrigenous runoff (Fig. 1) (*1*, *2*, *5*). As a result, shallow marine basins of western Tethys filled with sediment, causing seafloor topography to be flattened (*5*). Consequently, peritidal environments that now mark the base of the overlying Dolomia Principale (Hauptdolomit) carbonate platform (Fig. 1) extended for hundreds of kilometers (*25*). Oxygen-poor conditions were also common in some marine, semirestricted basins, as shown by the deposition of laminated shales in Italy, Austria, Hungary, Tunisia, India, and South China (Fig. 1) (*2*, *5*, *8*, *20*).

#### Terrestrial

The CPE had a major impact on terrestrial environments (Fig. 1). In the continental Central European Basin, a shift from playa-lake and continental sabkha environments to fluvial or freshwater lakes and littoral lagoons marks a change in the hydrological cycle (*1*, *26*). The Stuttgart Formation (Schilfsandstein) of Germany and equivalent units elsewhere are the expression of these large river systems that fed freshwater lakes, and large delta systems are seen throughout Europe (*27*). A delta that formed in the Norwegian Arctic reached its maximum extent at the time of the CPE and thus became the largest known delta system by area (1,000,000 km^2^) in Earth history (*28*). A complex palaeoenvironmental system, consisting of interlinked inland basins, developed along the North Atlantic rift system, extending from Greenland to Morocco, during a rifting phase (*26*, *29*, *30*). In these basins, the Carnian is characterized by lake sediments, with local increasing marine influence, coal deposits, and rivers that record wet climatic conditions but no widespread evaporites (*8*, *30*). In East Greenland, deep, freshwater lake or shallow marine sediments overlie fluvial or aeolian deposits (*31*), but more solid biostratigraphic age constraints are required for these deposits. In the Newark Basin of eastern North America, the Carnian is recorded in the Stockton Formation, which records giant fluvial systems and permanent, sometimes deep-water lakes (*29*, *32*). In Morocco, fluvial and lacustrine sedimentation replaces earlier arid sedimentation dominated by evaporites (*33*). In the Ischigualasto Basin in Argentina, radiometric dating and biostratigraphy show that a shift from fluvial to lacustrine deposition and then back to fluvial conditions occurred most likely during the CPE (*10*, *34*). Similar climatic shifts are seen from the coeval Santa Maria to Caturrita formations of Brazil (Fig. 1). In many sedimentary successions, fossil soils that formed during the CPE reflect the increase in rainfall, where analysis indicates that they developed under more humid conditions than seen either before and after (*2*, *35*, *36*).

### Carbon-isotope changes, global warming, and increasing precipitation

C-isotope (δ^13^C) records (*2*) show repeated perturbations of the global C cycle during the CPE [Fig. 1; see (*2*) for an in-depth discussion of the C-isotope records and how the composite curve was built correlating sections of the northwestern Tethys realm by using independent biostratigraphic constrains]. The first negative carbon isotope excursion (NCIE-1) is recorded by organic carbon (marine and terrestrial biomarkers and bulk organic matter) and bulk carbonate carbon at the onset of the CPE in the Tethys Ocean (*2*, *4*, *7*, *37*), the Boreal realm (*38*), and continental Pangea (*3*, *6*). NCIE-1 coincides with an initial pulse of siliciclastic material and the shift of the carbonate factory from predominantly microbial to metazoan reefs (*5*). Subsequent NCIEs are recorded in marine successions of Italy, Hungary, United Kingdom, and China (Fig. 1) (*2*, *6*, *7*) and, in northwestern Tethys, where they coincide with discrete siliciclastic influxes into the basins (*2*). In the Chinese successions, only one longer-term NCIE is recorded by organic matter in the interval, whereas carbonates show multiple excursions seen in other settings (*7*). The NCIEs are superimposed on an Anisian-Carnian positive δ^13^C trend, which is recorded by carbonates and organic matter (*4*, *5*). This trend has been attributed to the progressive increase of organic carbon burial linked to the re-emergence of coal swamps and peatlands after the early Triassic “coal gap” (*39*).

Isotope records indicate repeated injections of ^13^C-depleted carbon into the ocean-atmosphere system, which may have increased the pCO_2_ and likely triggered global warming (*2*). In the well-constrained continuous succession of the Nanpanjiang Basin in China (South China Block), where data also have high resolution, sea surface temperatures, derived from oxygen-isotope measurements on conodont apatite, indicate a possible two-pulse warming event in the Carnian, while other records from western Tethys show only one pulse (Fig. 1). In the successions of the South China Block, the onset of the CPE, namely, NCIE-1, coincided with the first pulse of warming of about 4°C. This was followed by a short cooling phase and then by a prolonged and more intense second phase of warming (*7*, *20*) starting from Tuvalian 1, matching the third NCIE, when temperatures increased by ca. 6°C. In the Lagonegro Basin, there is a major gap in the conodont δ^18^O record (Fig. 1), corresponding to the interruption of carbonate sedimentation in this deep-water succession, as discussed above. In the Northern Calcareous Alps, the temperature estimates from δ^18^O of conodonts show a warming during the CPE that peaks at the Julian-Tuvalian boundary (*40*), although the resolution of the data is lower than in the South China Block (*7*). This evidence suggests that the enhanced hydrological cycling during the CPE was linked to global warming, with increased evaporation leading to more continental runoff. In restricted marginal marine basins of Tethys, increased nutrient influx and, consequently, anoxia may have been responsible for the deposition of laminated shales (*5*).

## CARNIAN BIOLOGICAL CHANGES

### Marine ecosystems

**Fig. 2:**
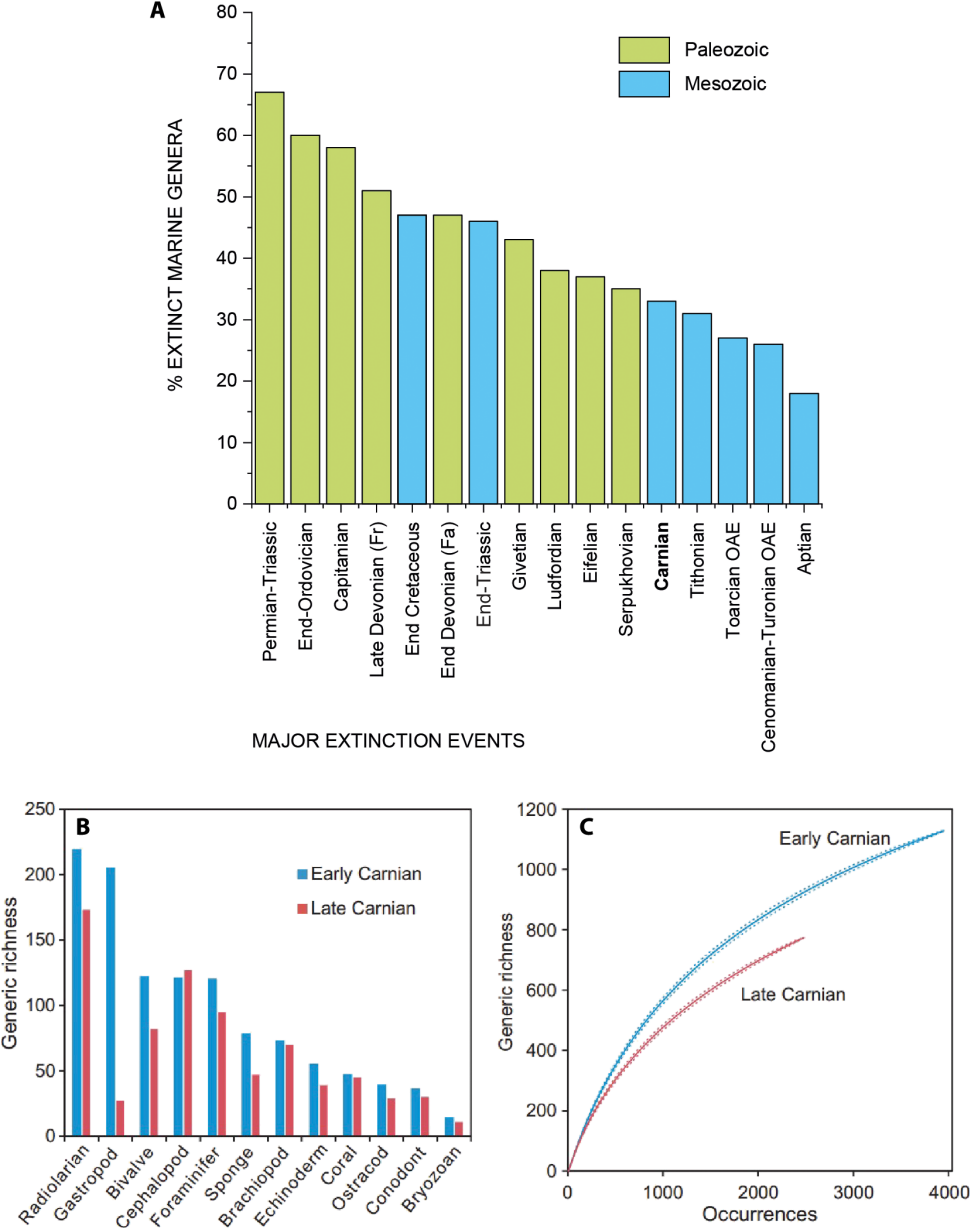
Marine extinctions during the CPE. (**A**) Comparison of extinction rates of all marine genera during the CPE with those of major Phanerozoic mass extinction events [from figure 2 of (*42*)]. The data compilation reveals that the CPE was a severe mass extinction, bigger than the well-known early Jurassic and Cretaceous extinctions. (**B**) Generic richness of 12 marine invertebrates’ groups for early and late Carnian. (**C**) Comparative rarefaction curves (full lines) with 95% confidence intervals (dash lines) for early and late Carnian. Rarefaction analyses were carried out on generic occurrences by using software PAST. Data come from the PBDB and the database of (*41*). OAE, Oceanic anoxic event.

Biodiversity data show a major turnover among marine invertebrates, with many of them suffering elevated extinction rates during the Carnian (Fig. 2) (*41*). The data of Sepkoski (*42*) show extinction of ~33% of diverse marine genera (invertebrates, vertebrates, and protists) during the Julian-Tuvalian boundary interval, i.e., within the CPE (Fig. 2A). Similarly, Bambach (*43*) noted higher levels of genus extinction in the Julian and the Tuvalian compared to other Mesozoic substages. Comparison with other extinction events shows that the Carnian extinction marks one of the largest marine loss of the Mesozoic (Fig. 2A). To better estimate the extinction severity and link this to the other observed phenomena, we analyzed the available fossil data from the Paleobiology Database (PBDB), whose ages and occurrences were revised, and from a revised global fossil database composed of 51,055 occurrences from 4221 collections in 1679 publications [see details in (*41*)]. This shows that marine invertebrate generic richness decreased from 1129 in the early Carnian (Julian) to 775 in the late Carnian (Tuvalian) (Fig. 2B). Rarefaction analysis shows that the decline of biodiversity is not an artifact of sampling (Fig. 2C). Most marine groups show a similar pattern, including the radiolarians, gastropods, bivalves, foraminifers, sponges, brachiopods, echinoderms, corals, ostracods, conodonts, and bryozoans (Fig. 2B). This decline, however, may be exaggerated for some groups because of variable sampling intensity. For example, early Carnian gastropods are known from over 600 occurrences, while there are only 37 late Carnian examples.

**Fig. 3:**
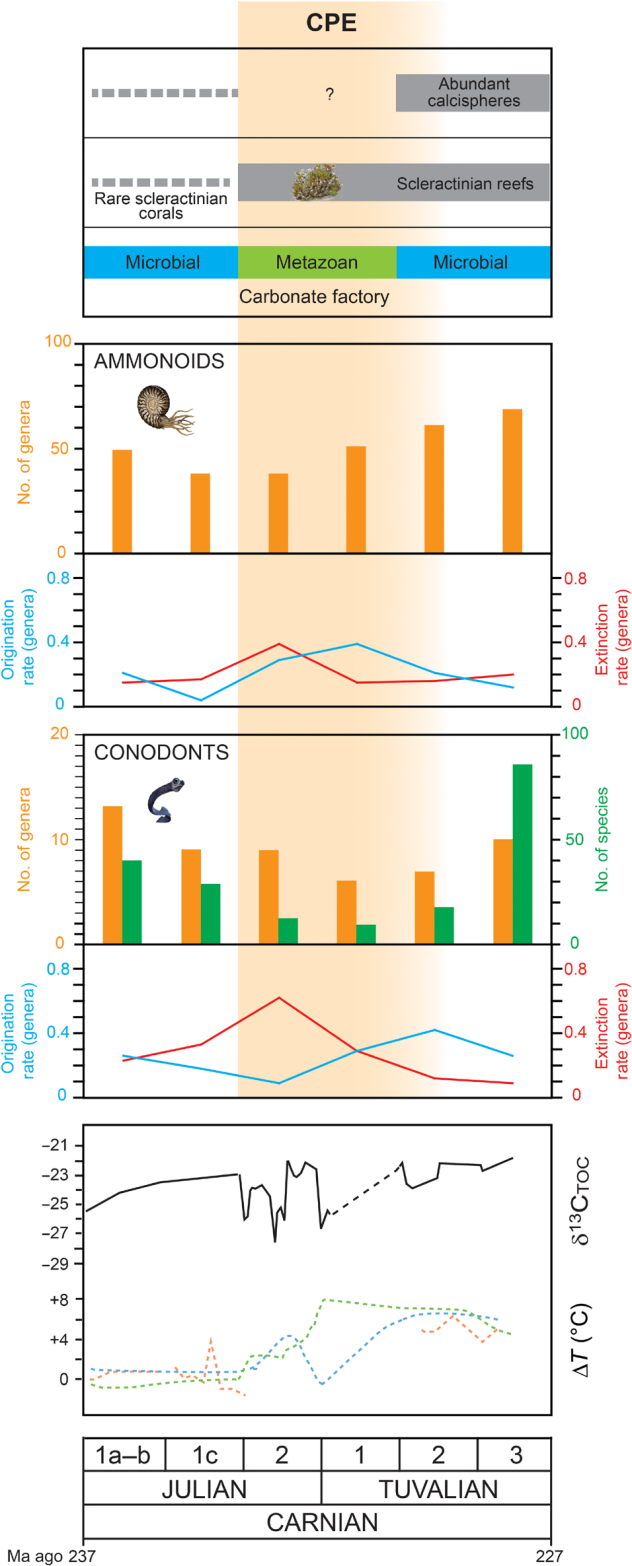
Marine extinctions and originations. The CPE marks the appearance of Scleractinian reefs and is followed by the first appearance of rock-forming calcareous dinoflagellates. Ammonoids and conodonts suffered a major extinction event at the onset of the CPE, followed by high origination rates in the Tuvalian. Extinction and origination rates are calculated according to (*114*), including singletons. Ammonoid and conodont data from the PDB have been revised and integrated. For the δ^13^C and T records, refer to Fig. 1.

High-resolution biodiversity data (at biozone level) can be compiled for ammonoids and conodonts, the principal biostratigraphic markers for the marine Late Triassic (Fig. 3). This allows better linking of extinction/origination events to the C-isotope record across the CPE. Although there was major provincialism among Late Triassic ammonoids (*15*), some were cosmopolitan, allowing fine time divisions of the Carnian. Ammonoid associations through the CPE include, in stratigraphic order, the cosmopolitan genera *Daxatina*, *Trachyceras*, and *Austrotrachyceras* (*17*). A major turnover occurred at the Julian-Tuvalian boundary, coincident with the third C-isotope excursion that marks the CPE, with high ammonoid extinction rates in the Julian 2 (earlier part of the CPE; Fig. 3) and high origination rates in the Tuvalian 1 (later part of the CPE; Fig. 3). The *Austrotrachyceras* faunas were suddenly replaced by new ammonoid groups, chiefly tropitids and juvavitids, which radiated rapidly during the late Carnian (Tuvalian) and produced diverse morphologies (*44*).

Conodonts also underwent a major turnover during the CPE (Fig. 3) (*11*, *45*), with a peak in extinction rates at the onset of the CPE, when diversity fell from 40 species in the early Julian to 13 species in late Julian times. Most early Carnian conodont taxa then disappeared across the Julian-Tuvalian boundary (*7*, *11*, *46*) with the lowest diversity of only nine species at the beginning of the Tuvalian (Fig. 3). They recovered after the CPE, but not as quickly as ammonoids, reaching precrisis levels only at the end of the Tuvalian (Fig. 3). After the CPE (Tuvalian 3, Fig. 3), conodont diversity was much higher than before the crisis.

Many crinoid groups, such as Encrinidae and Isocrinina (*1*, *47*), either went extinct or severely declined during the CPE. In general, benthic suspension feeders declined during the Carnian, as functional diversity analysis of the Late Triassic shows (*48*). In contrast, reef communities underwent a renaissance in the Carnian, and in some regards, this was the first step in the recovery of metazoan reef communities after they had been wiped out at the end of the Permian (*49*, *50*). Shallow-water reef-building colonial corals today are symbiotic with photosynthesizing dinoflagellate zooxanthellae, a relationship that first appeared in the Middle Triassic (*51*). However, initially, scleractinian corals were rare and accessory components of reefs. The earliest known Triassic examples of true coral reefs, or metazoan reefs in which corals were a key component (*49*, *52*–*54*), all date from the Carnian (Fig. 3). These changes are especially clear in western Tethys, where reefs from the Middle Triassic to early Carnian were constructed by microbes that formed up to 70% of framework carbonate (*23*). This microbial carbonate production reduced abruptly at the onset of the CPE, and the carbonate factory was replaced by carbonate ramps that hosted metazoan patch reefs with abundant scleractinian corals (*5*, *23*, *55*). This change in the carbonate factory is best seen in the Italian Dolomites, but it occurred worldwide, with evidence of similar changes from China (*7*), Turkey (*56*), and northern India (*20*). Microbial reefs returned in the Tuvalian (*2*), but the coral reefs that emerged during the CPE remained.

The CPE also marks the rise of calcareous nannofossils of possible dinoflagellate affinity (*Pithonella* group; Fig. 3) (*57*). Dinoflagellates probably originated in the Middle Triassic (*58*), but they only became widespread in the Carnian (*59*). These calcispheres are constructed from submicrometric calcite crystals, are found abundantly in deep-water late Carnian (Tuvalian) successions (*60*), and are extremely rare in older sediments (Fig. 3) (*60*, *61*). In post-CPE deep-water limestone in the Lagonegro Basin of Southern Italy, the calcispheres constitute ca. 10% of the total rock volume (*61*). By the end of the Triassic, 30 Ma later, the calcareous nannofossil *Prinsiosphaera* makes up >50% of rock volume in the Rhaetian pelagic chalks of Sicily (*62*). The rise of these calcispheres may represent a milestone in Earth history that could have fundamentally changed the global carbon cycle and certainly deserves more attention (see discussion below).

Marine Osteichthyes (bony fishes) suffered a major crisis during the CPE, when all groups experienced a decline in diversity of 51 to 62% (*63*). Osteichthyan communities of the Late Triassic were composed of palaeopterygians (large consumers) and subholosteans and neopterygians (small-sized consumers) (*63*, *64*). Neopterygians had begun to diversify in the Middle Triassic and radiated during the Late Triassic, with many evolving specializations for durophagy (*63*, *64*). Close outgroups of teleosts such as pachycormids and pholidophorids also radiated in the Late Triassic, but the diversification of true teleosts did not occur until the Late Jurassic.

Triassic sharks were composed largely of hybodonts, a group surviving from the Palaeozoic, and important throughout the Mesozoic, and the neoselachians, modern-type sharks. Neoselachians emerged in the Early Triassic but diversified in the Late Triassic, possibly during or slightly after the CPE (*63*), with seven genera becoming cosmopolitan and dominating shark-tooth faunas in Western Europe (*65*).

Marine reptiles may also have undergone a major turnover in the Carnian, although their record is incomplete. The early Carnian Guanling biota in China comprises diverse placodonts, ichthyosaurs, and thalattosaurs (*66*). There is then a gap until the Norian, when giant ichthyosaurs are known from North America. Thalattosaurs declined after the Carnian, and newly evolved clades, including the first plesiosaurs and parvipelvian ichthyosaurs, emerged probably in the late Carnian (*67*).

### Terrestrial ecosystems: Vertebrates

**Fig. 4:**
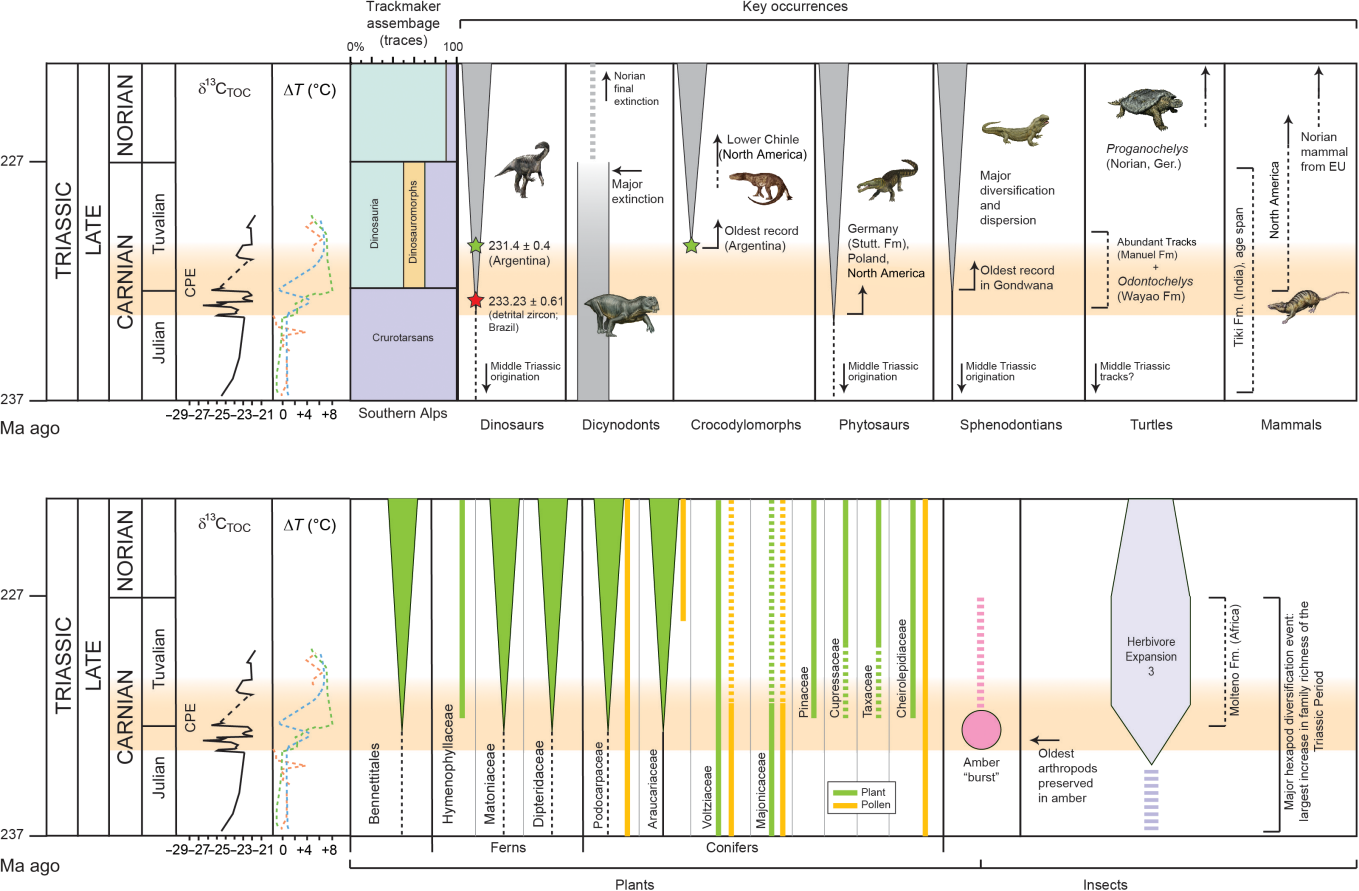
Terrestrial extinctions and originations. Major biological changes among vertebrates, plants, and insects, discussed in the paper, in relation to the C-isotope and *T* variations recorded during the Carnian (Fig. 1). The most notable pattern that can be observed is a major diversification event that affected many groups in the late Carnian (Tuvalian), i.e., just after the main negative C-isotope excursions that mark the CPE. Most of the amber, which indicates stressed conditions, is dated to the latest Julian when biostratigraphic data allow a good age control (*88*, *89*). Data are summarized from the following literature and the references therein. Trackmaker assemblage variations from (*10*) are as follows. Dinosauria: red star, detrital zircon age for Santa Maria Formation (Brazil); green star, U-Pb age of Ishigualasto fauna (*34*, *68*, *115*, *116*). Dycinodonts: (*117*, *118*). Crocodilomorpha: (*70*, *71*). Phytosaurs: (*119*, *120*). Rhynchocephalia: (*73*, *74*). Mammals: (*80*–*82*). Turtles: (*75*, *77*–*79*). Plants: (*83*). Amber: (*88*). Insects: (*91*–*93*). For the δ^13^C and T records, refer to Fig. 1. Stutt. Fm, Stuttgart Formation; EU, Europe; Ger, Germany.

The Carnian was a time of high extinction rates for several tetrapod clades (*14*, *16*), including the rhynchosaurs and dicynodonts, which were major herbivores of the time (Fig. 4). Revised dates show that the extinction of rhynchosaurs coincided with the CPE (*34*) in Argentina and Brazil, while dicynodonts underwent a major decline at the CPE, but as a group, they survived longer in some places, until at least 219 Ma ago (*68*).

Latest dating has confirmed a temporal link between the CPE and the Carnian dinosaur diversification event (*10*), which took place across Pangea right after the CPE. In the Dolomites of Northern Italy, dinosaur-dominated assemblages are found in the lithologic formation that records a switch back to arid conditions, which marks the end of the CPE (Fig. 4). This saw the expansion of saurischian dinosaurs and, ultimately, sauropodomorphs such as *Plateosaurus*, which became diverse and abundant in Germany, South America, and southern Africa, in the middle Norian, and later in North America (*10*, *68*, *69*). The dinosaur diversification event marks a major macroecological shift in tetrapod ecosystem structure at the time of the CPE, as suggested by both skeletal and footprint data (*14*).

The oldest crocodylomorphs, the clade including crocodilians, also appeared in the late Carnian (Fig. 4), with evidences by fossil occurrences from Argentina, Brazil, North America, and India (*70*, *71*). This is intriguing because it might imply a link between the diversification of both major archosaur groups (Dinosauria and Crocodylomorpha) and the CPE. Phytosaurs and rauisuchians were important carnivore groups, and both achieved a worldwide distribution during the late Carnian, with phytosaurs especially diversifying in the late Carnian (*72*).

Rhynchocephalia (lepidosaurs) seem to have experienced a major expansion event in the Carnian. The oldest known rhynchocephalian is from the Ladinian of Germany, Vellberg [Middle Triassic; (*73*)], but this group appears to have reached widespread distribution by the end of the Carnian as it is suggested by findings in very distant locations of Pangaea, in Brazil and the United Kingdom [see (*74*) and references therein].

The oldest basal turtle (*Odontochelys*), with a fully developed plastron (the ventral surface of the turtle shell) and a dorsal carapace composed of neural plates (early stages of carapace formation), was found in the Wayao Formation of China (*75*), of Carnian age (*7*, *76*). The next oldest turtles, which have fully developed shells (*Proganochelys*), are from the Norian of Germany (Keuper, Löwenstein Formation) (*77*). Ichnological studies suggest that the origin of turtles may date back to the Early Triassic (*78*, *79*). However, recent track findings in the Manuel Formation, which was deposited during the CPE (*26*), indicate that the new trophic resources and ecological niches from freshwater environments that developed during the CPE may have triggered the turtle radiation of the late Carnian (*79*).

The history of mammals also began in the Carnian (Fig. 4), with examples from India [Rewa Basin, Tiki Formation; (*80*)] and Texas (*81*). Mammals then diversified (but remained small and rare) in the Norian with morganucodonts, haramiyids, and “symmetrodonts” appearing in the United Kingdom, Germany, Greenland, and Luxembourg (*82*).

### Terrestrial ecosystems: Plants and insects

The Late Triassic floral record is sporadic, so changes in diversity can be tracked only at a coarse scale (*83*). Gondwanan macrofloras were dominated by corystosperm seed ferns, and pollen-spore assemblages were divided into a warmer northern Ipswich and a cooler southern Onslow flora (*83*). During the Carnian in Laurasia, there was a floral shift from arid-loving to humid-loving microflora (*21*, *83*) and macroflora (*83*, *84*), many associated with thick coal seams. The Carnian marks the full recovery from the so-called coal gap, the interval without formation of productive coal deposits that started with the collapse of the terrestrial ecosystems at the Permian-Triassic mass extinction (*85*).

The Carnian was an important period of radiation and diversification of several plant groups that would become important components of younger Mesozoic and modern floras. This includes the Bennettitales and several modern conifer families, the most prominent being the Cheirolepidiaceae (Fig. 4). Also, several modern fern families emerged and diversified during this time interval (Hymenophyllaceae, Matoniaceae, and Dipteridaceae) (*83*, *86*). Peltasperms and corystosperms reached their maximum diversity in the Carnian (*83*). In general, macroflora records show that these changes happened between the Julian (early Carnian) and Tuvalian (late Carnian) (Fig. 4), but the precise age requires better constraint.

An unusual aspect of the CPE in terrestrial settings is that it marks the first major finds of amber in the fossil record (Fig. 4) (*87*). This suggests widespread plant and terrestrial ecosystem stress (*88*), as a set of physical and chemical damages (e.g., storms and wildfire), insect outbreaks, and climate change toward moist conditions can all trigger an increase in resin production by conifers (*88*). Most Carnian amber is found between 5°N and 30°N latitude, in many localities worldwide. However, while the age constraints on the European amber occurrences are exceptionally good and indicate that this amber was actually formed during the CPE [especially those from the Dolomites; (*89*)], the precise age of some amber from other continents remains uncertain. South African amber found in the Molteno Formation (Karoo Basin) is Tuvalian (Fig. 4), but Triassic amber from Arizona (United States) is very likely younger, Norian in age [see discussion in (*89*)]. Amber droplets from the Dolomites in Italy contain the oldest organisms preserved in fossil resin (Fig. 4) (*89*). A prominent example is a previously unknown group of highly specialized, four-legged, phytophagous mites, belonging to the newly named superfamily Triasacaroidea, which is probably a sister group to the Eriophyoidea (extant gall mites) (*90*). Four distinct morphologies of these amber-preserved mites indicate a flourishing group during the CPE. The Carnian fossil record shows the first step in the evolution of modern herbivorous insects (Fig. 4) (*91*) and was part of a major diversification event (*92*) that included aquatic insects, hydraphagans (water beetles), and Staphylinidae (rove beetles). The herbivorous insect expansion in the Carnian is represented by the rich fauna of the Molteno Formation in the Karoo Basin (South Africa) (*93*). This formation is dated to the Tuvalian and is correlated to the Ishigualasto Formation (*94*), where the first body fossils of dinosaurs are also found. Many “modern” arthropod feeding modes, including piercing and sucking, galling, leaf mining, and seed predation seem to have spread in the Carnian (*93*, *95*).

### Extinction and emergence of new ecosystems during the CPE

Biodiversity data (*42*) of marine animals suggest a substantial reduction in generic and species richness in many different marine groups during the Carnian (Figs. 2 and 3). When high-resolution analysis is possible, it emerges that the marine extinction occurred mainly during the late Julian, with high extinction rates among ammonoid and conodonts, while high origination rates are recorded in the early Tuvalian (Fig. 3). Qualitative data suggest a roughly similar picture for the terrestrial realm, where the main diversifications also appear to have occurred in the Tuvalian (Fig. 4). Hence, the data indicate that the CPE can be the “smoking gun” that caused widespread Carnian extinctions, and this event was followed, in the Tuvalian, by a remarkable explosive diversification of important groups that are now key components of modern ecosystems. In the seas, these new ecosystems included the first modern-style reefs, abundant rock-forming calcispheres, new mollusk groups, and durophagous fishes. On land, we observe the diversification of several modern conifer and fern families as well as the Bennettitales and tetrapod groups such as archosaurs, turtles, crocodiles, and mammals (Fig. 4).

The flourishing of metazoan (coral) reef communities is indeed a remarkable characteristic of the CPE when compared to other extinction events. During the end-Triassic extinction, coral reefs were severely damaged and fully recovered only in the middle Jurassic (*96*). Similarly, the Permian-Triassic event was marked by the most severe reef crisis of the Phanerozoic, when the Palaeozoic metazoan reef ecosystems went extinct (*54*). Both the Permian-Triassic and the end-Triassic reef crises have been linked to ocean acidification (*97*). On the contrary, the CPE seems to have been marked by changes in the carbonate cycle that somehow favored calcifying organisms. In this respect, the coeval rise of calcareous nannofossils (of possible dinoflagellate affinity) is also remarkable but requires more in-depth research to fully comprehend the taxonomy of these organisms, their paleogeographical extent and their abundance to understand their possible role in switching CaCO_3_ production from neritic environments into deeper waters (*61*). The formation of deep-water carbonate deposits in the Mesozoic introduced an additional carbonate reservoir that helped stabilize the global carbon cycle by enhancing the buffering capacity of the ocean (*98*). Hence, the rise of these still enigmatic calcispheres and their remarkable abundance in deep-water deposits, which begins during the CPE, could have been the first step of the “mid Mesozoic revolution” in ocean chemistry driven by pelagic calcifiers (*98*).

The effects of the CPE on reshaping terrestrial ecosystems are probably comparable to those of the Cretaceous Terrestrial Revolution (KTR). During the KTR, terrestrial biodiversity exceeded that in the sea, and the angiosperms rose to ecological dominance (*99*): The radiation of angiosperms probably triggered the diversification of many lineages of insects, birds, mammals, and seed-free land plants and fungi (*100*).

Summarizing, the CPE can be seen as the dawn of a Mesozoic marine and terrestrial revolution that, through the emergence of major evolutionary innovations and profound changes in global biogeochemical cycles, resulted in the shaping of modern ecosystems (*101*). Our review also highlights limitations in our understanding of the Carnian biological changes. On the one hand, dating of the terrestrial records is sometimes not yet robust enough, and most age estimates provide only a general (early and/or late) Carnian age, which cannot be precisely linked to the CPE and its multiple geochemical and environmental shifts. On the other hand, marine fossils, which are potentially much better constrained in terms of age, appear less studied than terrestrial ones, especially for groups other than conodonts and ammonoids.

## WRANGELLIA LIP VOLCANISM

**Fig. 5:**
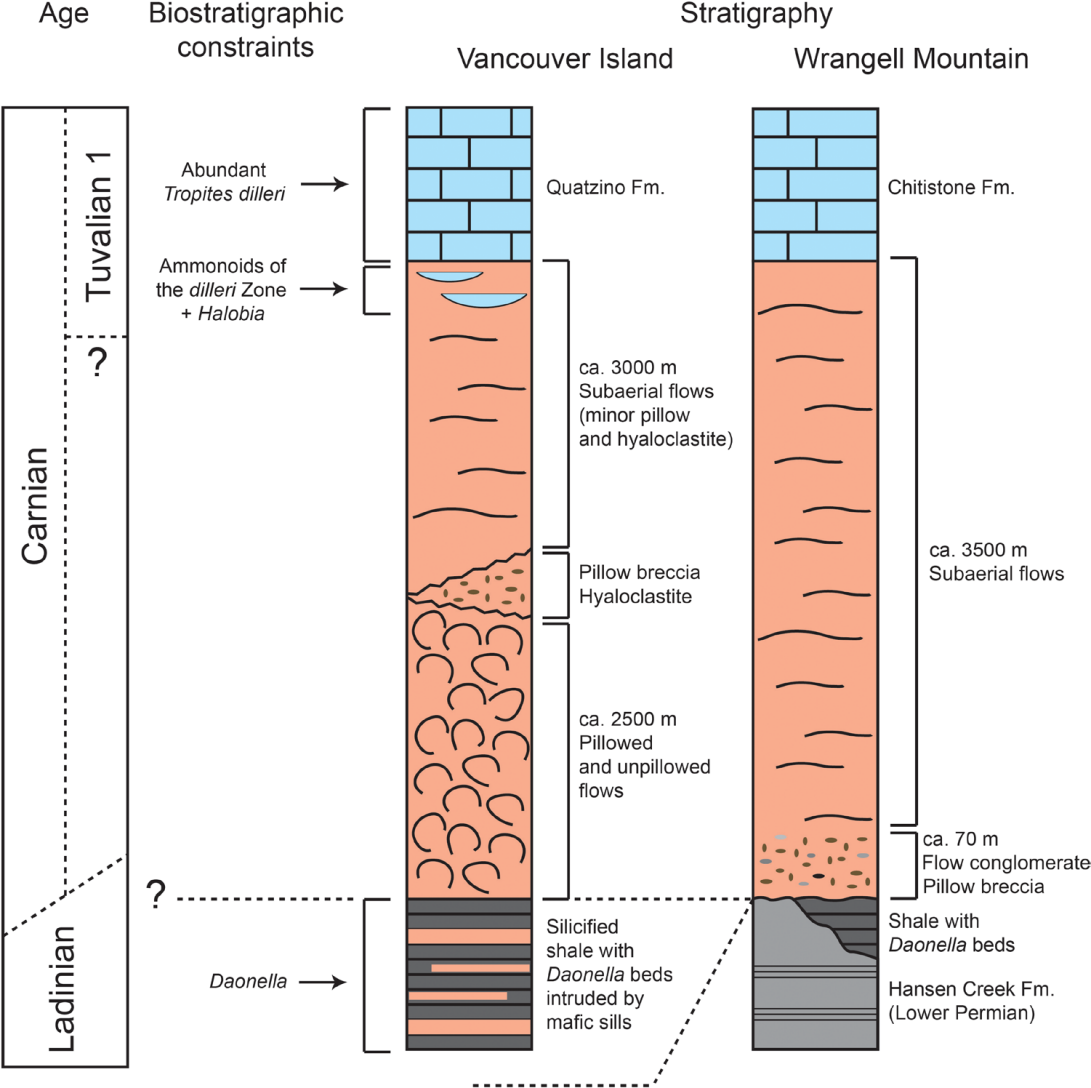
Wrangellia LIP. Stratigraphy of the Wrangellian succession in Vancouver Island (Canada) and Wrangell Mountain (Alaska, USA) reconstructed from (*102*). The age of the end of Wrangellia volcanism can be solidly fixed with ammonoid biostratigraphy to the early Tuvalian, while the onset could have a late Ladinian age given the presence of *Daonella* bivalves below the basalt pile (see discussion in the main text). Fm., Formation.

Biostratigraphic data show that the eruption of the Wrangellia LIP occurred during the Carnian and its age, at least partially, overlaps with the age of the CPE [Fig. 5; (*4*, *9*, *102*)]. This LIP erupted at equatorial latitudes in eastern Panthalassa (Fig. 1), accreted during the Late Jurassic–Early Cretaceous, and today outcrops in northwestern North America (*102*). The Wrangellia basalt succession is typically 3.5 to 6 km thick, but the original volume of the erupted basalts is difficult to calculate because much could have been subducted during the Late Jurassic–Early Cretaceous accretion. Estimates from geological mapping suggest that at least 1 million km^3^ of basalts were erupted (*102*). Part of the basalts was erupted under water, but a large part of the volcanic sequence (especially the upper part) is subaerial (Fig. 5). Interbedded with, and above, the last basalt flows of Wrangellia (Karmutsen basalt), ammonoids belonging to the *dilleri* Zone (Tuvalian 1) are found in Vancouver Island [Fig. 5; (*103*, *104*)]. In Frederick Island of the Queen Charlotte Island, the Wrangellian volcanics (Karmutsen basalt) are overlain by the Kunga limestone (i.e., Sader, Peril, and Sandilands formations). The lowermost Sader Formation is mainly of Tuvalian age (probably Tuvalian 2 and 3) based on conodont biostratigraphy (*105*). Below the first basalts, *Daonella* bivalves, which are Ladinian in age, are present on Vancouver Island and Wrangell Mountain (Fig. 5). Where the contact is exposed, there is a disconformity-unconformity between the Wrangellia basalts and the underlying sediments (*106*); for example, at Wrangell Mountain (Alaska; Fig. 5) below the Nikolai Formation (the local name for the Wrangellia basaltic pile), the sediments belong to the Hansen Creek Formation of early Permian age (*106*). Hence, while the end of Wrangellia LIP volcanism appears to be well constrained biostratigraphically to the early Tuvalian by marker ammonoids, the onset is less well known and could be latest Ladinian–early Carnian on the basis of fossil contents. Os isotope records for the Middle-Late Triassic support a possible latest Ladinian onset of Wrangellia volcanism (*107*), but we need higher resolution as well as more robust age constraints (*5*). Existing data show that at least the late part of Wrangellia volcanism (where *dilleri*, Tuvalian 1 ammonoids are found, Fig. 5) is certainly coeval with the CPE (Julian 2 to Tuvalian 1 in age, Fig. 1).

Radioisotopic ages for Wrangellia basalts are scarce. Available ^40^Ar/^39^Ar age spectra are affected by the widespread alteration of Wrangellia basalts, with apparent ages ranging from the Carnian to the Cenozoic and analytical errors of up to 11 Ma ago (*102*, *108*). The two available U-Pb ages on baddeleyte or zircon for this LIP are also not very robust, as they were obtained on chemically untreated multigrain aliquots unlike the high-quality single zircon ages available for other LIPs (*109*).

Other Tethyan volcanic events occurred during the CPE (Huglu-Pindos Series, Kare-Dere basalts, and South Taimyr Complex) that could also have also played a role in triggering the observed changes (*7*), but their volumes and exact age span must be determined to evaluate their contribution. We reiterate our earlier statement that most Triassic ocean crust has now been destroyed by subduction, meaning that the survival of Wrangellia tends to focus attention on this LIP, but the possibility remains that other magmatic/volcanic eruptions could have occurred in the Carnian.

In Earth history, episodes of LIP magmatism frequently coincided with major climate changes and biological turnovers, including the Permo-Triassic and end-Triassic mass extinctions (*110*). LIP volcanism can account for pulsed and relatively quick injection of large quantities of CO_2_ into the atmosphere-ocean system. Calculations suggest the Wrangellia eruptions released at least 5000 gigatons of C, and the greenhouse effect can explain the temperature rise and enhancement of the hydrological cycle during the CPE (*4*). The C-isotope excursions that punctuate the CPE cannot be explained by volcanic CO_2_ alone because it is not isotopically light enough (δ^13^C = −6‰). As hypothesized for other LIP-related events, release of additional ^13^C-depleted CO_2_ from ocean floor methane clathrates (δ^13^C = −60‰) and/or organic-rich sediments (δ^13^C = −35 to −50‰) could have increased the greenhouse effect and contributed to the NCIE (*2*). Global warming (*7*) and possible oceanic anoxia (*2*) are detrimental to marine life and could have caused deep ecosystem perturbations (*110*). Besides CO_2_, LIP volcanism can emit large quantities of SO_2_, halogens such as Cl and F, and highly toxic elements like Hg, Zn, and Cd, and other gases (*110*), as reported for the end-Triassic extinction (*111*). SO_2_ can cause short-term cooling through formation of sulfate aerosols in the stratosphere and acid rain (sulfuric acid) on reaction with water. Acid rain affects terrestrial ecosystems by acidifying soils, lakes, and streams and damages plants and aquatic animals (*112*). LIP-related SO_2_ damage on plant cuticles have been described for fossils from end-Triassic beds of Greenland (*113*).

As mentioned above, Wrangellia LIP volcanism probably ended at the base of the Tuvalian 2 (Fig. 5) and could have been most active in the Julian. It is also likely that some basalt was still being erupted in the latest phase of the CPE, since the ammonoid *Tropites dilleri*, whose distribution falls entirely within the Tuvalian 1, was found in sedimentary deposits intercalated between basalts in Vancouver Island (*102*). Wrangellia volcanism could therefore explain why high extinction rates and major environmental changes are observed mainly in the latest Julian [Figs. 3 and 4; (*2*)] and how the extinction and environmental change could have resulted in the consequent Tuvalian radiation by making new ecological niches available. However, because of the lack of precise stratigraphic and geochronological links between Wrangellia and the CPE, we can only speculate on the possible volcanic triggers for the observed extinctions and environmental changes by analogy with other LIP-related events.

## PERSPECTIVE

Paleontological data suggest that the CPE was a major (but previously neglected) time of extinction and may be linked to the Carnian explosive diversification of many key modern groups of plants and animals. Evidence indicates a possible cascade of events similar to other mass extinctions: LIP eruption as the trigger, release of volcanic gases, rapid shifts in temperature and δ^13^C, ocean anoxia, and major ecosystem remodeling characterized by both extinctions and diversifications, coupled to milestone changes in carbonate systems, with the emergence of scleractinia coral reefs and the rise of calcispheres as rock-forming components in deep-water settings. The extensive turnover of life that appears to be linked to the CPE ended the phase of instability started by the Permian-Triassic mass extinction and marked the emergence of modern ecosystems. Both the beginning and end of this phase of biological revolution can be linked to the emplacement of LIPs, showing the importance of these massive volcanic events in shaping the biosphere. However, many aspects of the CPE remain unclear and poorly studied:

1) Stratigraphic resolution needs improvement through additional radioisotopic dates and magnetostratigraphy to better constrain the timing of extinction and radiation and their temporal link with the CPE. Existing data indicate that most of the observed biological changes actually happened during the CPE, but for many groups, especially for terrestrial fauna and flora, the age uncertainty is still too large to define a precise link with this event.

2) Records of carbon cycling would benefit from additional δ^13^C records across the CPE, from both continental and marine settings, and from different latitudes.

3) Reconstructions of atmospheric pCO_2_ are needed through the CPE.

4) Records of anoxia, nutrient feedbacks, and sulfur cycling are sparse or absent; hence, state-of-the-art geochemical techniques like Fe and P speciation, S-isotope analysis, and trace metal analysis could help to better define and constrain the complex series of biogeochemical phenomena that mark the CPE.

5) Improving the resolution of the existing palaeobiology databases like the PBDB. The CPE extinction is a within-stage event that is difficult to capture given the current resolution of data stored in the major databases: Our meta-analysis shows that a rigorous and detailed revision of the PBDB entries and improvement of resolution (at biozone level in the case of ammonoids and conodonts) actually reveal the major turnover that marks the CPE and that previously was only qualitatively defined.

6) It is crucial to better constrain the age of the Wrangellia eruptions with improved radioisotopic dating to precisely fix the onset of the LIP volcanism and its duration and calculate the rates at which the magma was erupted and the gases were released into the atmosphere-ocean-land system. More geochemical data (e.g., S-isotopes and Hg) are also needed to trace Wrangellia volcanism in the sedimentary record and therefore understand the causal relationships with the observed biological changes.

Integrating detailed stratigraphic, geochemical, and radioisotope data will clarify the sequence of events and possible cause-and-effect relationships, so helping in understanding this unique interval in Earth history and the intimate causes of the extinction and, maybe most crucially, the unique diversification event that led to the formation of modern ecosystems. This will also put the CPE in the framework of long-term changes that characterize the entire Triassic. Last, understanding the complex interactions between volcanism, environmental, and biological changes during the CPE might reveal crucial information about the mechanisms that initially controlled the Mesozoic transition toward the modern Earth system.
